# Effects of flunarizine combined with ginkgo leaf extract and dipyridamole injection on hemorheology in elderly patients with vertigo

**DOI:** 10.12669/pjms.40.3.8456

**Published:** 2024

**Authors:** Xinlei Liu, Lingyu Shu, Junxia Zheng

**Affiliations:** 1 Xinlei Liu, Department of Neurology, The First People’s Hospital of Lin’an District, Hangzhou, Zhejiang Province 311300, P.R. China; 2 Lingyu Shu, Health Management Center, The First People’s Hospital of Lin’an District, Hangzhou, Zhejiang Province 311300, P.R. China; 3 Junxia Zheng, Health Management Center, The First People’s Hospital of Lin’an District, Hangzhou, Zhejiang Province 311300, P.R. China

**Keywords:** Flunarizine, Ginkgo leaf extract and dipyridamole injection, Hemorheology, Vertigo

## Abstract

**Objective:**

To investigate the effect of flunarizine combined with ginkgo leaf extract and dipyridamole injection (GDI) on hemorheology of elderly patients with vertigo.

**Methods:**

Clinical data of 105 elderly patients with vertigo who were treated in The First People’s Hospital of Lin’an District from June 2019 to December 2022 were retrospectively selected. Of them, 54 patients received flunarizine combined with GDI (Study group) while 51 patients received flunarizine treatment alone (Control group). The treatment effect and adverse reactions of the two groups, functional rehabilitation before and after treatment, including the Simplified Vertigo Symptom Score Scale (VSS-SF), Berg Balance Scale (BBS), and Dizziness Handicap Inventory (DHI) were measured. Hemodynamics including blood flow velocity (Vm) of basilar artery (BA), left vertebral artery (LVA), and right vertebral artery (RVA) before and after treatment were also assessed.

**Results:**

The total efficacy of the treatment in the study group was higher than that in the control group (94.4 % *vs*. 75.9%; P<0.05). After the treatment, the Vm of the BA, LVA, and RVA was increased in both groups compared to before treatment, and the increase was greater in the study group than in the control group (P<0.05). In addition, the BBS scores of the two groups after the treatment were higher than before the treatment, while the DHI and VSS-SF scores were lower than before the treatment. BBS scores of the study group were higher than those of the control group, while the DHI and VSS-SF scores were lower than those of the control group (P<0.05). There was no statistically significant difference in the incidence of adverse reactions between the study group (5.6%) and the control group (2.0%; P>0.05).

**Conclusions:**

The combination of flunarizine and GDI in elderly patients with vertigo can effectively regulate hemodynamics of the patient, reduce the degree of vertigo, improve balance, and have a significant overall therapeutic effect without increasing the risk of adverse reactions.

## INTRODUCTION

Vertigo is the sensation of movement caused by abnormal spatial orientation. A lifetime prevalence of vertigo ranges from 3-10%.^1^ and the incidence of this condition increases significantly with age. Current data show that approximately 30% of elderly people may experience vertigo.^2^ Advanced age leads to longer hospitalization times and is associated with significant financial burden on patients and the healthcare system.^3^ Therefore, vertigo can greatly impact patient’s daily life and overall physical and mental health.^3^,^4^

Flunarizine and ginkgo leaf extract, and dipyridamole injection (GDI) are all important drugs in the clinical treatment of cerebrovascular diseases.^5^,^6^ Flunarizine can improve cerebral blood flow by maintaining intracellular ion regulation mechanisms, quickly restoring brain blood supply, and alleviating hypoxia and ischemia.^5^ GDI is a compound preparation of dipyridamole, ginkgo flavone glycosides, and terpene lactones which can improve cerebral ischemia symptoms, expand cerebral blood vessels, and protect the circulatory. cardiovascular and cerebrovascular systems.^6^

While research has been conducted to investigate the effectiveness of GDI,^7^ there is still limited data on the effectiveness of the combination of flunarizine and GDI. Thus, this study aimed to conduct a retrospective analysis of the clinical data of elderly vertigo patients treated with flunarizine combined with GDI in our hospital to explore the clinical effectiveness and safety of flunarizine combined with GDI in the treatment of elderly patients with vertigo.

## METHODS

Clinical records of 105 elderly patients with vertigo who were treated in The First People’s Hospital of Lin’an District from June 2019 to December 2022 were retrospectively selected for this study. The cohort included 62 males and 43 females with an average age of 73.0 ± 7.2 years. Fifty-four patients were treated with flunarizine combined with GDI and were assigned to the study group, while 51 patients were treated with only flunarizine and were considered the control group.

### Inclusion criteria:

- Conforming to the diagnostic criteria for vertigo.^8^
- Accompanied by varying degrees of loss of balance, nausea, limb numbness, dizziness
- Age ≥ 60 years old.
- Brain CT examination revealed vertebrobasilar artery insufficiency.

### Exclusion criteria:

- Vertigo caused by neck injury.
- Patients with primary cerebral vascular malformations.
- Patients taking other drugs during treatment.
- Those with other cardiovascular and cerebrovascular diseases.
- Vertigo caused by epilepsy, demyelinating disease, encephalitis, cerebral hemorrhage, or cerebral infarction.
- Patients with malignancy.
- Patients with incomplete data.

### Ethical Approval

This study was approved by the Medical Ethics Committee of the First People’s Hospital of Lin’an District, Hangzhou (No.2023-14, date: 2023-03-30).

### Control group

Patients received flunarizine (Shanxi Zhendong Ante Biopharmaceutical Co., Ltd., Approval No.: H14020844) monotherapy. Flunarizine was administered orally twice a day, 10 mg/time. Once symptoms had subsided, the dose was changed to oral administration twice a day, at 5 mg/time. The medication was taken for 14 days.

### Study group

Patients received flunarizine combined with GDI. On the basis of flunarizine treatment, patients in the study group also received GDI (Guizhou Yibai Pharmaceutical Co., Ltd., Approval No.: H52020032) by intravenous drip containing 20 ml GDI+250 ml physiological saline once a day. The course of the treatment was 14 days.

### Treatment indicators:

### Treatment effect

If the VSS-SF score decreased by ≥ 70%, and the symptoms of nausea, limb numbness, and dizziness completely disappear, it was considered a significant effect. If the VSS-SF score decreased by 30% to 69%, and there was some relief of nausea, limb numbness, and dizziness, it was considered effective. If the VSS-SF score decreased by less than 30%, and nausea, limb numbness, dizziness, and other symptoms do not subside, it was considered invalid. Total efficacy = (significant effect + effective)/total number of cases × 100%.

### Hemodynamics

Blood flow velocity (Vm) of basilar artery (BA), left vertebral artery (LVA), and right vertebral artery (RVA) was measured using the Aloka SSD 1700 Doppler ultrasound (Aloka Ltd, Tokyo, Japan).

### Functional rehabilitation effects

Each patient’s functional rehabilitation was evaluated using the Simplified Vertigo Symptom Score Scale (VSS-SF), Berg Balance Scale (BBS), and Dizziness Handicap Inventory (DHI). The VSS-SF has a total of 60 points, and the higher the score, the more severe the dizziness symptoms.^9^ The BBS has a total of 56 points, the higher the score, the stronger the balance ability.^10^ The DHI has a total of 100 points, and the higher the score, the more severe the degree of dizziness.^11^

### Adverse reactions

Adverse reactions such as rash, vomiting and nausea, and gastrointestinal reactions were recorded during treatment for both groups.

### Statistical Analysis

SPSS 25.0 (SPSS Inc., Chicago, IL, USA) was used for data analysis. Categorical variables were expressed as number of cases and compared via Fisher’s exact test or Chi-squared tests. Continuous variables were expressed as means ± standard deviation (SD) and compared via Student’s t-tests. Significance was set at *p* <0 .05.

## RESULTS

There were 29 males and 22 females in the control group, with an average age of 72.45 ± 7.25 years. The average course of disease in the group was 3.24 ± 1.20 years, and the average body mass index was 23.33 ± 2.63kg/m^2^. In the study group, there were 33 males and 21 females with an average age of 73.52 ± 7.22 years, an average course of disease of 3.02 ± 1.21 years and average body mass index of 23.91 ± 2.60 kg/m^2^. There were no significant differences in baseline patient characteristics such as gender, age, course of disease, and body mass index between the two groups (*P*>0.05; Table-I). The total efficacy of treatment in the study group was 94.4% which was higher than 75.9% in the control group (*P*<0.05; Table-II).

**Table-I:** Comparison of baseline characteristics.

Group	n	Gender (Male/Female)	Age (year)	Course of disease (year)	BMI (kg/m^2^)
Study-group	54	33/21	73.52±7.22	3.02 ± 1.21	23.91±2.60
Control-group	51	29/22	72.45±7.25	3.24 ± 1.20	23.33±2.63
*χ^2^*/*t*		0.124	0.756	-0.919	1.151
*P*		0.725	0.451	0.360	0.252

**Table-II:** Comparison of treatment effects between the two groups [n (%)].

Group	n	Significant effect	Effective	Invalid	Total effective
Study-group	54	29(53.7)	22(40.7)	3(5.6)	51(94.4)
Control-group	51	22(40.7)	19(35.2)	10(18.5)	41(75.9)
*χ^2^*					4.774
*P*					0.029

Before the treatment, there was no statistically significant difference in the Vm of the BA, LVA, and RVA between the two groups (*P*>0.05). After the treatment, the Vm of the BA, LVA, and RVA was increased in both groups compared to before the treatment, and the increase was greater in the study group compared to the control group (*P*<0.05; Table-III).

**Table-III:** Comparison of hemodynamics between the two groups (*χ̅*±s, cm/s).

Time	Group	n	BA Vm	LVA Vm	RVA Vm
Before treatment	Study-group	54	23.61±3.05	22.26±3.10	20.22±4.19
	Control-group	51	24.22±3.15	21.72±3.24	19.94±4.51
	t		0.999	0.862	0.331
	P		0.320	0.391	0.741
After treatment	Study-group	54	39.81±3.86^a^	28.22±3.56^a^	30.44±4.91^a^
	Control-group	51	35.62±3.97^a^	25.59±3.54^a^	25.82±4.67^a^
	t		5.482	3.792	4.942
	P		<0.001	<0.001	0.001

***Note:*** Compared with this group before treatment,

a P<0.05.

Before the treatment, there was no statistically significant difference in the scores of BBS, DHI, and VSS-SF between the two groups (*P*>0.05). After treatment, there was an increase in the BBS score in both groups compared to before treatment, while the DHI and VSS-SF scores were lower compared to before treatment. Moreover, the BBS score in the study group was higher than the control group, while the DHI and VSS-SF scores were lower than those in the control group (*P*<0.05; Table-IV). There was no statistically significant difference in the incidence of adverse reactions between the study group (5.6%) and the control group (2.0%) (P>0.05; Table-V).

**Table-IV:** Comparison of functional rehabilitation effects between the two groups (*χ̅*±s, points).

Time	Group	n	BBS	DHI	VSS-SF
Before treatment	Study-group	54	20.37±3.85	61.07±6.15	28.02±3.50
	Control-group	51	19.59±3.81	59.88±6.49	27.04±4.32
	t		1.045	0.966	1.279
	P		0.298	0.336	0.204
After treatment	Study-group	54	45.07±4.63	13.04±3.76	9.15±2.95
	Control-group	51	38.41±4.13	18.16±4.87	12.76±2.90
	t		7.766	-6.003	6.622
	P		<0.001	<0.001	<0.001

***Note:*** Compared with this group before treatment, ^a^P<0.05.

**Table-V:** Comparison of adverse reactions between the two groups [n (%)].

Group	n	Rash	Vomiting and nausea	Gastrointestinal reactions	Total occurrence rate
Study-group	54	1(1.9)	0(0)	2(3.7)	3(5.6)
Control-group	51	0(0)	1(2.0)	0(0)	1(2.0)
*χ^2^*					0.925
*P*					0.336

## DISCUSSION

This study showed that flunarizine combined with GDI is significantly more effective than flunarizine alone and can also improve hemodynamic indicators of elderly patients with vertigo. Our study further showed that the functional rehabilitation scores related to vertigo symptoms improved following flunarizine treatment alone, but the improvements were more pronounced in the study group.

Meng S et al.^12^ showed that simple Flunarizine treatment of vertebrobasilar insufficiency vertigo can alleviate the symptoms, regulate the hemodynamic status of vertebrobasilar artery, and improve the quality of life of patients. However, the overall effectiveness of medication alone still need to be improved. Li Jian et al.^13^ and Cun R et al.^14^ used flunarizine and ginkgo dipyridamole injection to treat vertigo patients, and showed that this combination had a good therapeutic effect compared with ginkgo dipyridamole treatment alone. Our study is consistent with these results.

Flunarizine administration in patients with vestibular migraine and vertigo can alleviate the degree of vertigo and pain to a certain extent, which may improve patients’ quality of life.^15^ There is also evidence to support the use of flunarizine and topiramate in patients with vestibular migraine to reduce the frequency, intensity, and duration of vertigo, and also regulate their emotional state.^16^ Moreover, no serious adverse events occurred during the treatment period, confirming that flunarizine is safe in the treatment of vertigo and vestibular migraine.

Flunarizine and GDI are widely used in the treatment of neurological diseases.^5^,^6^ Many clinical studies have shown that flunarizine is a selective calcium antagonist, which can maintain the intracellular ion regulatory mechanism, avoid calcium overload in cells, reduce nerve excitability, alleviate and eliminate the continuous depolarization caused by vascular smooth muscle contraction, reduce the degree of vertebral artery spasm, inhibit cerebral vasoconstriction, and restore normal blood supply to the vertebral artery, thereby reducing the degree of hypoxia and ischemia in the brainstem vestibular system.^17^-^19^ Studies have indicated that many patients with vertigo have varying degrees of vertebrobasilar artery hemodynamic abnormalities, which can exacerbate the degree of hypoxia and ischemia in brain tissue.^19^,^20^ After a large amount of calcium ions flow in, this can cause calcium overload, leading to neuronal damage. Flunarizine can effectively penetrate the blood brain fluid barrier, prevent excessive calcium ions from entering the membrane, and thereby alleviate the degree of vertigo caused by insufficient blood supply.^20^,^21^ On the other hand, GDI can alleviate cerebral ischemia, improve memory, inhibit cell aging, clear oxygen free radicals, and regulate the microcirculation.^5^,^22^ The main components of GDI include ginkgo damo extract, which contains ginkgolides, flavonoids, and dipyridamole.^23^ Dipyridamole can inhibit platelet aggregation, downregulate vascular wall permeability, regulate arterial vasodilation, and restore hemorheology. It can also promote vasodilation while reducing blood viscosity, inhibit excessive aggregation of platelets and red blood cells, strengthen the regulatory effect of red blood cells on heart and cerebral blood vessels, gradually restore cellular structure, strengthen the protective ability of neural function, and promote neurotransmitter formation and nerve conduction 24. GDI can effectively regulate vascular tension, improve cerebral hemorheology, two-way regulate and antagonize platelet activating factors, alleviate the degree of hypoxia and ischemia, protect nerves, and relax vascular smooth muscle.^23^

In addition, Cao et al.^25^ have shown that the ester components in GDI are specific antagonists of platelet activating factor receptors, which can inhibit platelet aggregation and thromboxane A2 production caused by platelet activating factor, reduce blood viscosity, and avoid thrombosis. Furthermore, the xanthone component can regulate peripheral vascular resistance, enhance vascular elasticity, and tension, and increase blood flow, thereby improving cerebral microcirculation and alleviating the degree of dizziness. Therefore, GDI and flunarizine can synergistically increase cerebral blood flow, alleviate cerebral vasospasm, improve blood circulation in the brain, alleviate dizziness, and enhance treatment effectiveness.

### Limitations

This was a single center, retrospective analysis, with a small sample size. It is possible that there may be selection bias. In addition, only a small number of disease indicators was analyzed in this study. Moreover, the study included patients who received flunarizine as a control group. A comparison with GDI alone is suggested in future studies. Finally, there was no follow-up component to the study, and an understanding of the long-term effects of the combined treatment in elderly vertigo patients is warranted.

## CONCLUSION

The combination of flunarizine and GDI in the treatment of elderly patients with vertigo can effectively regulate the hemodynamic state, reduce the degree of vertigo, improve balance, and have a significant therapeutic effect without increasing the risk of adverse reactions. The combined medication may be a promising therapeutic drug for elderly patients with vertigo. It should be further validated through future studies and its application in clinical practice should be explored.

### Authors’ contributions:

**XL:** Conceived and designed the study.

**LS** and **JZ:** Collected the data and performed the analysis.

**XL:** Was involved in the writing of the manuscript and is responsible for the integrity of the study.

All authors have read and approved the final manuscript.
